# miR-24 functions as a tumor suppressor in Hep2 laryngeal carcinoma cells partly through down-regulation of the S100A8 protein

**DOI:** 10.3892/or.2011.1571

**Published:** 2011-11-30

**Authors:** YAN GUO, WEINENG FU, HONG CHEN, CHAO SHANG, MING ZHONG

**Affiliations:** 1 Department of Medical Genetics, China Medical University, Shenyang 110001; 2 Department of Central Laboratory, School of Stomatology, China Medical University, Shenyang 110007; 3 Department of Neurobiology, China Medical University, Shenyang 110001, P.R. China

**Keywords:** laryngeal squamous cell carcinoma, miR-24, *S100A8*, invasion, translational regulation

## Abstract

microRNAs, a family of small non-coding RNAs, regulating approximately 30% of all human genes are deeply involved in the pathogenesis of several types of cancers, including laryngeal squamous cell carcinoma (LSCC). Here, we demonstrate that miR-24 is down-regulated in human LSCC tissues. Ectopic expression of miR-24 in Hep2 cells significantly induced cell morphology changes and inhibited cell proliferation and invasion ability *in vitro* by targeting *S100A8* at the translational level. Meanwhile, miR-24 could significantly inhibit Hep2 cell invasion after S100A8 protein blockade. In conclusion, our results suggest that miR-24 may function as a tumor suppressor in LSCC through down-regulation of *S100A8*, which suggests that miR-24 could serve as a novel potential maker for LSCC therapy.

## Introduction

Laryngeal squamous cell carcinoma (LSCC) is one of the most common head and neck cancers in the world. Now, the main treatment strategy for LSCC is still surgery or total laryngectomy followed by radiotherapy. For the majority of patients with advanced cases, existing scheme may seriously impair their laryngeal function and further affect the quality of life (1). Although genetic and epigenetic alterations were systematically analyzed to guide improvement in survival rates and treatments, the molecular mechanisms leading to LSCC development and progression are complex and not entirely clear.

A new dimension of gene regulation mechanisms has been identified since the discovery of microRNAs (miRNAs), which are small non-coding single stranded RNAs of ~22 nucleotides in length, which negatively regulate gene expression by repressing translation or decreasing the stability of mRNAs depending on the degree complementarity between the miRNA and its target (2,3). Recent evidence indicated that miRNAs have important roles in many biological processes, including cell differentiation, proliferation and apoptosis (4). Altered miRNA expression profiles are involved in various types of human cancers and seem to function as oncogenes and tumor suppressors by targeting mainly the 3'un-translation region (3'UTR) of associated genes (5–7). Although miRNAs have been predicted to regulate approximately 30% of all human genes, few miRNAs have been assigned to their target mRNAs and specific functions.

Most evidence suggested that miR-24 is aberrantly expressed in many kinds of cancers and the erythropoiesis process, which lead to significantly malignancies and cell differentiation alteration (8–11). Of clinical significance, miR-24 is involved in the regulation of oral squamous cell carcinoma (OSCC) growth and that the expression level of miR-24 in plasma might be validated as a tumor marker for OSCC (12). Another study showed that miR-24 emerged as a biomarker specific for Kaposi sarcoma (13). However, the relationship of miR-24 to LSCC is not yet reported. In this study, we explored the role and mechanism of miR-24 in the development and aggression of LSCC by analyzing the biological characteristics and regulation manner of miR-24 in LSCC.

## Materials and methods

### Materials, antibodies, cell lines and patient tissues

Media/FBS were purchased from Invitrogen/Gibco (Karlsruhe, Germany), pGL3-Promoter vector from Promega (Madison, WI, USA), control miR and pre-miR-24 from Ambion (Austin, TX, USA), Lipofectamine™ 2000 from Invitrogen (Carlsbad, CA, USA), and Transwell chambers (1 cm^2^, 12 mm pores) from Machery-Nagel (Düren, Germany). Power SYBR-Green PCR Master Mix was obtained from Applied Biosystems (Foster City, CA, USA). S100A8-antibody was purchased from Santa Cruz Biotechnology (Santa Cruz, CA, USA), and β-actin-antibody was from Sigma. Hep2 (human laryngeal cancer) and HEK293 (human embryonic kidney) cell lines were acquired from the Cell Biology Institute of Shanghai, Chinese Academy of Science. Tissue specimens (tumor tissues, matched non-tumor tissues) from 20 patients with LSCC were collected after the patients gave informed consent. Verification of the specimens was performed by a pathologist and the samples were immediately frozen at −80˚C.

### miRNA and gene expression analysis

microRNA and total RNA were isolated using the mirVana™ miRNA isolation kit (Ambion, TX, USA) according to the manufacturer's instructions. The concentrations of small and total RNA were measured by reading the absorbance at OD260/280 nm. microRNA cDNAs were synthesized with the QuantiMir RT Kit Small RNA Quantitation System (System Biosciences, CA, USA). Briefly, microRNA templates were polyadenylated followed by ligation to an oligo-dT adaptor and reverse transcriptions were then performed to generate small RNA cDNAs. Total RNA cDNAs were synthesized from 1 μg of total RNA in the presence of oligo-dT (12–18) primer (Takara, Japan) and MMLV reverse transcriptase according to the manufacturer's instructions (Promega).

Expression of miR-24 was determined by the SYBR-Green-based real-time quantitative PCR (qPCR). U6-snRNA was used as internal control. A miR-24 specific primer and a universal reverse primer RTQ-UNIr were used for the amplification. The qPCR was performed with Power SYBR-Green PCR Master Mix in a 30 μl reaction volume on the Applied Biosystems 7500HT. All primer sequences used for *S100A8* and miR-24 detection are listed in Table I. PCR reactions were performed at 95˚C for 10 min, followed by 40 cycles of 95˚C for 15 sec and 60˚C for 1 min. ΔCt was calculated by subtracting the Ct of U6 or β-actin mRNA from the Ct of the mRNA of interest. ΔΔCt was then calculated by subtracting the ΔCt of the negative control from the ΔCt of the samples. The fold change in mRNA or microRNA was calculated according to the equation 2^−ΔΔCt^.

### Cell morphology, in vitro proliferation and invasion assay

The Cell-based experiments were carried out by transfection of 50 nM pre-miR-24 or control miR into Hep2 cells using Lipofectamine™ 2000 in accordance with the manufacturer's procedure. Cell morphology was evaluated at day 7 after transfection by ×10 objective magnification using an Eclipse TE2000-S microscope (Nikon).

For cell proliferation analysis, 2–3×10^3^ Hep2 cells after transfection were plated into 96-well plates in triplicate. Cells were then cultured for 1, 3, 5 and 7 days. The absorbance at 570 nm was measured after incubation the cells with 100 μl sterile MTT dye (0.5 mg/ml, Sigma) for 4 h at 37˚C and 150 μl DMSO for 15 min. Then the cell growth curve was constructed by using OD570 nm as ordinate axis.

In the invasion assay, cells transfected with microRNA precursor were transferred to the upper compartment (2×10^5^ cells/well) of Transwell chamber in 25 μl serum-free medium. The supernatant of mouse NIH3T3 (500 μl) was added to the bottom compartment. Following incubation for 12 h at 37˚C, cells invaded into the lower surface of the membrane were fixed and stained with haematoxylin and eosin. Then cell numbers in 5 randomly selected fields were counted under a light microscope.

### microRNA target prediction

The prediction of S100A8 mRNA as a target of microRNAs was made with miRanda (http://cbio.mskcc.org/cgi-bin/mirnaviewer/mirnaviewer.pl?type=miRanda) and RNA22 (http://cbcsrv.watson.ibm.com/rna22.html) software. The default stringency settings included the maximum number of allowed UN-base paired bases (equal to 0 in seed/nucleus of 7 nucleotides), the minimum number of paired-up bases in heteroduplex (equal to 14) and the maximum folding energy for hetero-duplex (225/Kcal/mol).

### 3'UTR-luciferase plasmid construction and reporter assays

The full-length 3'UTR of S100A8 gene was amplified using cDNA from Hep2 cells (primer sequences are listed in Table I). Then, the PCR product was cloned into the *Xba*I-site of pGL3-Promoter vector, checked for orientation, sequenced and named Lut-S100A8-Wt. Site-directed mutagenesis of the miR-24 target site in the S100A8 3'UTR (Lut-S100A8-Mut) was carried out using the Quikchange Mutagenesis Kit (Stratagene, Heidelberg, Germany), with Lut-S100A8-Wt serving as a template (mutagenic oligonucleotide primer sequences are listed in Table I.

For reporter assays, Hep2 cell were transiently co-transfected with luciferase plasmid and microRNA precursor (control miR: 5′-UGGAAUGUAAAGAAGUAUGUA-3′, pre-miR-24: 5′-UGGCUCAGUUCAGCAGGAACAG-3′) using Lipofectamine™ 2000. Reporter assays were performed after 36 h post-transfection using the Dual Luciferase assay system (Promega), normalized for transfection efficiency by co-transfecting renilla luciferase. The group of cells transfected with control miR and mutant reporter plasmid served as negative control for specificity. Each experiment was conducted in triplicate.

### Cell lysate preparation and Western blot analysis

After transfection of the luciferase plasmid and miRNA precursor, Western blot analysis was performed as previously described (14). Briefly, cells were washed with PBS and lysed in RIPA buffer. Protein concentration was determined by BCA (Pierce, IL, USA). Aliquots (25 mg) were separated on 12% SDS-PAGE and transferred to PVDF membrane. The membrane was then blocked and incubated with S100A8 antibody (1:1000) followed by horseradish peroxidase-conjugated antibody (1:5000). Detection was performed by enhanced chemi-luminescence (ECL) using a Western blotting luminological reagent (Santa Cruz Biotechnology) according to the manufacturer's instructions. β-actin was used as a reference protein, and was determined following the same procedure as above.

### Statistical analysis

All values were reported as the means ± standard deviation. Differences were assessed by one-way analysis of variance (ANOVA) and Student's unpaired t-test using software SPSS 13.0. P<0.05 was considered to be statistically significant.

## Results

### miR-24 is down-regulated in human LSCC specimens

**Figure 1:**
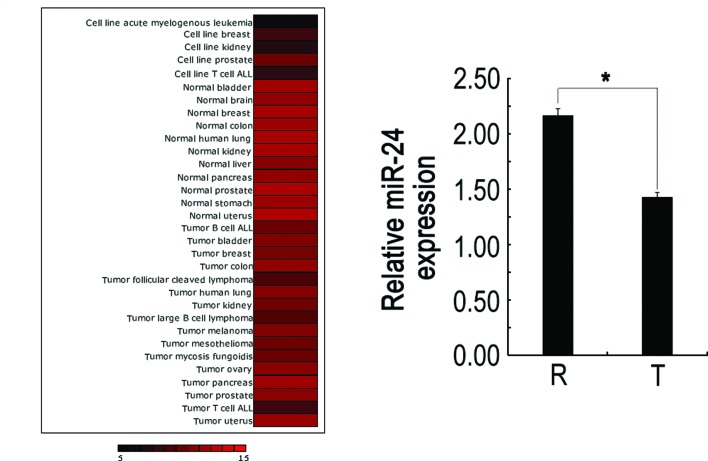
The expression of miR-24 is down-regulated in LSCC tissues. (A) We searched the database of miR-24 expression in normal and cancer tissues by miRNAMap-2.0, and found that miR-24 was usually up-regulated in normal cell lines/tissues while was down-regulated in cancer cell lines/tumor samples, as compared to the controls. (B) Quantitative analysis of the expression levels of miR-24 normalized to those of U6 by qRT-PCR. T, tumor tissues; R, matched non-tumor tissues. ^*^P<0.05.

It has been observed that miR-24 was aberrantly expressed in human malignancies, including tongue squamous cell carcinoma. In addition, we investigated miR-24 expression in normal vs. cancer tissue using miRNAMap-2.0 (15), and found that miR-24 levels were frequently up-regulated in normal but down-regulated in tumor samples (Fig. 1A). To explore the possible role of miR-24 in LSCC development, we tested miR-24 expression in LSCC obtained from 20 patients by SYBR qRT-PCR. As shown in Fig. 1B, 75% (15 of 20) of carcinoma tissues showed reduced miR-24 expression with respect to normal counterparts, and the average expression level in carcinoma tissues was significantly lower than that in normal larynx tissues, which is consistent with the miRNAMap-2.0 gene chip results. Together, these results suggest that miR-24 plays an important role in LSCC development.

### miR-24 induces morphological change and impairs proliferation and invasion properties in Hep2 cells

**Figure 2:**
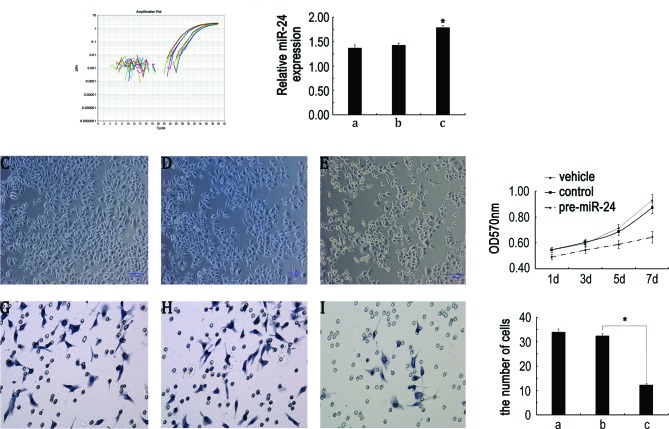
miR-24 induces morphological changes and impairs proliferation and invasion properties in Hep2 cells. (A) Amplification plot of miR-24. (B) Statistical analysis of miR-24 expression. a, vehicle group; b, control miR transfection group; c, pre-miR-24 transfection group. (C–E) Representative images showing the morphology of Hep2 cells transfected with vehicle, control miR and pre-miR-24, respectively. Scale bar, 100 μm. (F) Cell proliferation was measured by the MTT assay. Results are means of three independent experiments ± SD. (G–I) Representative images of invasive cells on the membrane by transfection vehicle, control miR and pre-miR-24 for 7 days, respectively (magnification, ×400). (J) Statistical analysis of average invasive cell number of three independent experiments ± SD (^*^P<0.05). a, vehicle group; b, control miR group; c, pre-miR-24 group.

Given that miR-24 is markedly down-regulated in laryngeal carcinoma, it may thus function as a potential tumor suppressor. To investigate whether miR-24 down-regulation played a causative role in Hep2 cells, we assessed the biological effect of miR-24 on the development and/or progression of LSCC using a gain of function approach. As shown in Fig. 2A and B, qRT-PCR revealed that miR-24 precursor (pre-miR-24) enhanced miR-24 level, suggesting that pre-miR-24 is efficiently introduced into the cells and the following detection is invincible. Microscope observed result showed that transfection of Hep2 cells with pre-miR-24 resulted in morphological changes, including the increase of round-shaped cells and the reduction of cell number compared with control groups (Fig. 2C–E). MTT assay showed that the cell proliferation ability displayed a time-dependent tendency among the three groups. Especially on day 7, the cells transfected with pre-miR-24 showed significantly lower proliferation ability than those in control groups (Fig. 2F). The above results indicate that the expression level of miR-24 has an influence on cell growth *in vitro*.

Meanwhile, we assessed the effects of miR-24 on cell invasion, a key determinant of malignant progression and metastasis. As shown in Fig. 2G–I, the invasion effect of pre-miR-24 on Hep2 cells by Transwell showed the number of trans-membrane Hep2 cells undergoing pre-miR-24 transfection was much lower than those in control groups on day 7. The transmembrane Hep2 cell number ranged from 34±1.25 and 32.48±0.95 to 12.37±0.52 in the vehicle, control miR and pre-miR-24 transfection groups, respectively. ANOVA analysis showed a significant statistical difference between the pre-miR-24 transfection and control groups (P<0.05, Fig. 2J), which implies that down-regulation of miR-24 may contribute to tumor metastasis in LSCC.

### S100A8 mRNA is a target of miR-24

**Figure 3:**
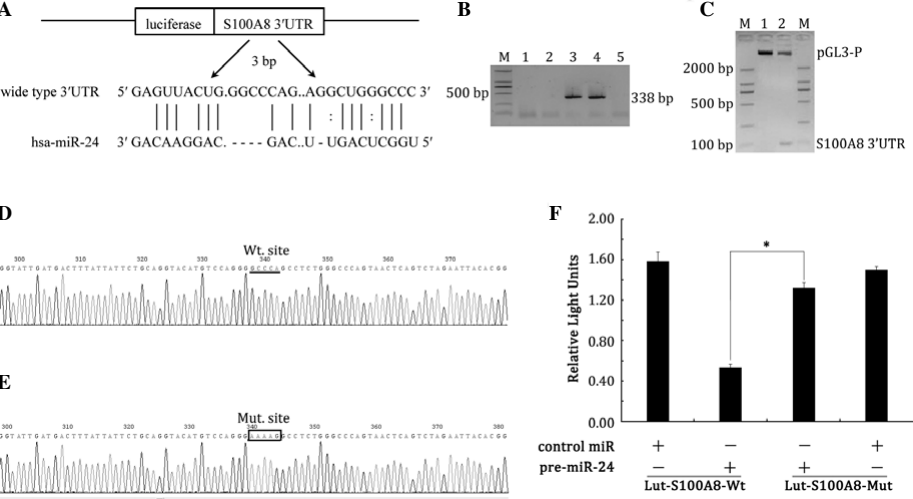
*S100A8* is a validated target of miR-24. (A) 3'UTR of *S100A8* is predicted to be a target of miR-24. (B) PCR screening result of Lut-S100A8-Wt construct. (C) Enzyme digestion result of Lut-S100A8-Wt construct. M, DL 2000 marker; 1, pGL3-promotor circular plasmid; 2, Lut-S100A8-Wt digested by *Xba*I. (D) Sequencing result of Lut-S100A8-Wt construct. (E) Sequencing result of Lut-S100A8-Mut construct. Rectangular box, mutated site. (F) The reporter assay, with each bar representing values from three independent experiments performed in quadruplicates. The transfection efficiency was normalized by co-transfected renilla luciferase and the light units were calculated by relative luciferase activity of firefly to renilla. ^*^P<0.05.

As miR-24 has a pivotal function in LSCC, the question how the miRNA exerts its role in LSCC needs to be investigated. In this study, *S100A8* mRNA was predicted to be a potential target of miR-24 after computational analysis using two different programs (miRanda and RNA22). The programs returned a hit between the 455 bp sequences of *S100A8* and miR-24 (Fig. 3A).

To confirm whether the 3'UTR of *S100A8* was a functional target of miR-24 in LSCC, we set up a luciferase reporter assay. The wild-type of *S100A8* gene 3'UTR, harboring miR-24 target sites, was cloned into the downstream of pGL3 plasmid, which was driven by the SV40 basal promoter, Lut-S100A8-Wt. PCR screening, enzyme digestion and sequencing identified that Lut-S100A8-Wt had the correct origin, right sequence compared with GeneBank database, with no frame shift (Fig. 3B–D). In parallel, we cloned a second reporter construct, named Lut-S100A8-Mut, in which the conserved targeting site of miR-24 was specifically mutated, putatively to abolish the miRNA binding ability. Fig. 3E showed that the Lut-S100A8-Mut plasmid was constructed successfully. Then the Hep2 cells (which have low endogenous miR-24 expression) were co-transfected with the reporter vector and miRNA mimics. As shown in Fig. 3F, Hep2 cells with Lut-S100A8-Wt and pre-miR-24 led to a significant decrease in reporter activity compared to the controls (P<0.05). However, the activity of the reporter construct with a mutation at the miR-24 target site was unaffected when Hep2 cells were co-transfected with pre-miR-24 (P>0.05). Together, these results indicate that 3'UTR of *S100A8* carries a direct and specific binding site of miR-24 *in vitro*.

### miR-24 down-regulates S100A8 expression at translational level

**Figure 4:**
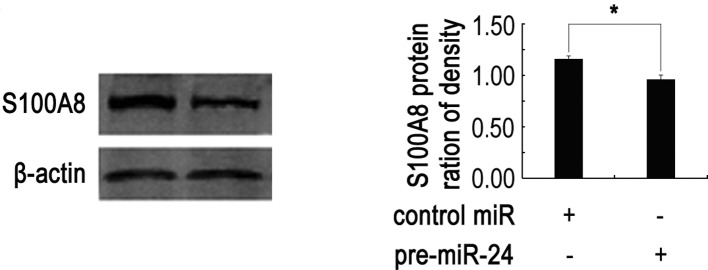
S100A8 protein levels in Hep2 cells detected by Western blotting. (A) Representative image of the protein level of S100A8. β-actin was used as a reference control. (B) Quantitative analysis of the relative protein levels of S100A8 normalized to those of β-actin is shown. Data are the mean ± SD of three independent experiments. ^*^P<0.05.

miRNAs can regulate gene expression through decreased translation of target mRNA, increased degradation of target mRNA, or both. To test at which level *S100A8* was down-regulated by miR-24, qRT-PCR and Western blotting assays were performed. Compared with controls, miR-24 had no significant effect on the *S100A8* mRNA expression (data not shown), while the S100A8 protein level was significantly down-regulated when Hep2 cells were cotransfected with miR-24 and Lut-S100A8-Wt (P<0.05) (Fig. 4). These results strongly suggest that miR-24 negatively regulates *S100A8* expression through translational repression pathways.

### miR-24 inhibits Hep2 cell invasion partly through S100A8 blockage

*S100A8* gene is one of the important inflammation factors, which has the ability to promote tumor cell invasion. Therefore, we further down-regulated endogenous S100A8 protein level using S100A8 antibody blocking, and then detected the role of *S100A8* in miR-24 mediated Hep2 cell invasion after cells were transfected with pre-miR-24 36 h. Transwell results showed that the transmembrane cells of pre-miR-24 and control miR transfection groups were 12.37±0.52 and 32.48±0.95, respectively. The transmembrane cells of S100A8 protein that blocked following pre-miR-24 transfection group was much less, with only some cells observed, and no more than 15 cells in the whole membrane. ANOVA analysis showed significant statistic difference among the groups, P<0.05. This result indicates that S100A8 protein plays a critical role in miR-24 mediated Hep2 cell invasion.

## Discussion

miRNAs are essential regulators of many cellular processes including proliferation, differentiation, metastasis, and morphogenesis. They are usually overexpressed or show loss of expressed in almost all carcinogenesis and tumor development. Moreover, half of these aberrant miRNAs located in the genetic region of oncogenes or tumor suppressor genes, functions as proto-oncogenes or tumor suppressors. Substantial evidence shows that miR-24 encoding gene maps to human chromosome 9q22 and 19p13, regions that are unstable and frequently altered in head and neck squamous cell carcinoma (16,17).

Several studies have shown that miR-24, functioning as a tumor suppressive gene, plays an important role in human cancer and other diseases. For example, Mishra *et al* found that miR-24 was abnormally down-regulated in human colorectal cancer tumors and showed a p53-independent cellular proliferation (8). Rogler *et al* showed that miR-24 was up-regulated in hematopoietic carcinoma and induced tumor suppressive activities (7). However, Liu *et al* reported the up-regulation of miR-24 in tongue squamous cell carcinoma (TSCC) and the miR-24 mediated changes led to enhanced proliferation and reduced apoptosis in TSCC cells (19). Although the expression of miR-24 in cancers is controversial, the functional evidence for a role of miR-24 has been documented consistently. Our results show that the up-regulation of miR-24 leads to significant cell morphological changes, reduced cell number, low proliferation and enhanced cell invasion potential of LSCC, which implies that miR-24 is associated with the biological effect of LSCC and retards the development of LSCC. Importantly, studies have clarified the high stability of miRNAs in blood and the increase in plasma miR-24 is detectable in patients with a low level of miR-24 up-regulation in tumors (12,20). Since the sampling of blood is relative non-invasive and the examination of blood can facilitate early diagnosis, our findings further reveal that plasma miR-24 might be a potential useful LSCC biomarker.

The discrepancy of miR-24 expression and function might be mainly due to its effect on the multiple target genes (2,21). Recent studies found that *FAF1, DHFR, E2F2, MYC* and other cell cycle regulatory genes are target genes of miR-24 (8,9,21,22). However, given that a single miRNA has multiple targets, we believe that miR-24 also has other targets. Computational algorithms have been the major driving force in predicting miRNA targets (23). Through analysis using miRanda and RNA22, we identified *S100A8* as a possible target of miR-24 among the regulated genes. Further, we used series of experiments to confirm that *S100A8* is a direct negative target gene of miR-24 in LSCC. First, overexpression of miR-24 significantly reduces the luciferase activity of 3'UTR sequence of *S100A8*, while mutation at the miR-24 target site in the 3'UTR of *S100A8* could significant decrease the miR-24 regulation effect. These results indicate that *S100A8* 3'UTR carries a direct and specific miR-24 binding site. Second, over-expression of miR-24 significantly down-regulates S100A8 protein expression, while has no effect on *S100A8* mRNA level, which further suggests that miR-24 directly regulate *S100A8* gene through translational level.

As a new target gene of miR-24, S100A8 is an important inflammation factor, localized in the cytoplasm and/or nucleus of a wide range of cells. S100A8 and S100A9 are often co-expressed as a complex, suggesting a common mechanism of transcriptional regulation in inflammatory diseases and cancers (24). Accumulating evidence suggests that infection and inflammation contribute to 15–20% of all malignancies. Recent clinical and experimental data have suggested that changes in the expression and/or function of the S100A8 protein may represent a key step during cancer development (25,26). Yao *et al* concluded that overexpression of *S100A8* is associated with stage, progression, invasion, metastasis and poor survival in human bladder cancer (27). Additionally, obvious up-regulation of *S100A8* has been found in breast, lung, gastric, colorectal, pancreatic and prostate, while down-regulation of *S100A8* has been detected in squamous esophageal carcinomas (24). Our previous research also validated the up-regulation of *S100A8* in LSCC, leading to Hep2 cells invasion and enhanced metastasis ability (28). Collectively our present results indicate that miR-24 down-regulated in LSCC leads to loss of tumor suppressor function, which enhanced Hep2 cell invasion. As a direct target of *S100A8*, miR-24 negatively regulates *S100A8* expression at translational level. We speculate that miRNA-24 inhibits LSCC Hep2 cell invasion through regulating S100A8 protein expression.

Additionally, previous studies have found that down-regulation of *S100A8* via RNA interference could promote apoptosis and inhibit metastasis in LSCC (28). In the present study, we found that miR-24 acts as an endogenous siRNA for *S100A8*, and cell invasion ability induced by *S100A8* can be subtracted by the overexpression of miR-24. Thus, the identification of *S100A8* as a miR-24 target gene provides a possible explanation as to why the overexpression of miR-24 can inhibit LSCC Hep2 cell invasion.

In conclusion, miR-24 was down-regulated in LSCC, and functions as a tumor suppressor in Hep2 cells partly through targeting the *S100A8* gene. Our findings revealed that the identification of miR-24 and its target gene *S100A8* in LSCC may help to understand the molecular mechanism of carcinogenesis, and also give us strong rationale to further investigate miR-24 as a potential treatment target for LSCC.

## Figures and Tables

**Table I:** Primer sequences used in the present study.

Gene	Primer sequence
miR-24	Specific primer: F: 5′-TGGCTCAGTTCAGCAGGAACAG-3′ RTQ-UNr: CGAATTCTAGAGCTCGAGGCAGGCGACATGGCTGGCTAGTTAAGC TTGGTACCGAGCTCGGATCCACTAGTCC (T) 25VN
*S100A8*	F: TTGCTAGAGACCGAGTGTCC R: CTTTGTGGCTTTCTTCATGG
*S100A8* 3'UTR	F: 5′-GAATCTAGACTGAGTTACTGGGCCCAGAG-3′ R: 5′-CTTCTAGAGAGGTATTGATGACTTTATTAT-3′
Mutagenic *S100A8* 3'UTR	F: 5′-GAGTTACTGGGCCCAGAGGCCTTTTCCCTGGACATGTACCTGCAG-3′ R: 5′-CTGCAGGTACATGTCCAGGGAAAAGGCCTCTGGGCCCAGTAACTC-3′
